# Late Follow-up of Patients Submitted to Total Cavopulmonary Derivation: Clinical Aspects, Reinterventions, and Complications Interfering in Morbidity and Mortality

**DOI:** 10.21470/1678-9741-2017-0217

**Published:** 2018

**Authors:** Cristiane Felix Ximenes Pessotti, Paula Rodrigues Silva Machado Costa, Natalia de Freitas Jatene Baranauskas, Thalyta Madeira Correa, Ieda Biscegli Jatene

**Affiliations:** 1 Hospital do Coração (HCor), São Paulo, SP, Brazil.

**Keywords:** Fontan Procedure, Heart Defects, Congenital, Treatment Outcome, Heart Ventricles/Pathology

## Abstract

**Objective:**

To identify main complications in outpatient follow-up, as well as factors before or during operation that may interfere in patient's evolution.

**Methods:**

Retrospective study of patients submitted to total cavopulmonary shunt with extracardiac conduit from 2000 to 2014 at the Hospital do Coração (São Paulo, Brazil) and who underwent clinical follow-up at this institution.

**Results:**

One hundred and fifty surgeries were performed and 59 patients maintained outpatient follow-up. The mean age of these patients at the time of surgery was 4.45 years (median of 45 months) and 70.2% of them were males. Among the patients undergoing outpatient follow-up, postoperative time at evaluation ranged from 10 days to 145 months; 30 (50.8%) patients had single left ventricle and 29 (49.2%) had single right ventricle (48.2% of these presented with hypoplastic left heart syndrome [HLHS]). Patients with single left ventricle had a higher percentage of reintervention-free survival, but without statistically significant difference. 40% of the patients had no complications and 35% of them presented with thrombosis at some point in the follow-up period, with ventricular dysfunction being the second most frequently found complication (15% of cases), mainly among patients with single right ventricle morphology (*P*=0.04). Between the patients currently under follow-up, 20 (35%) of them had been evaluated by ultrasonography and had some degree of hepatic congestion and/or hepatomegaly. 16.7% of the patients with such alteration had HLHS (*P*=0.057).

**Conclusion:**

Except for the right ventricular morphology, no other factor has been shown to interfere in late evolution after total cavopulmonary shunt.

**Table:** 

Abbreviations, acronyms & symbols				
ACE	= Angiotensin converting enzyme		PAIVS	= Pulmonary atresia with intact ventricular septum
ASA	= Acetylsalicylic acid		PLE	= Protein-losing enteropathy
CAVSD	= Complete atrioventricular septal defects		PTFE	= Polytetrafluoroethylene
DILV	= Double inlet left ventricle		RV	= Right ventricle
DORV	= Double outlet right ventricle		RVOT	= Right ventricular outflow tract
EC	= Extracardiac conduit		SPC	= Systemic-to-pulmonary collateral
HLHS	= Hypoplastic left heart syndrome		SV	= Single ventricle
LV	= Left ventricle		TAPVD	= Total anomalous pulmonary venous drainage
MPAP	= Mean pulmonary artery pressure		USG	= Ultrasonography
PA	= Pulmonary atresia		VSD	= Ventricular septal defects

## INTRODUCTION

Single ventricular hearts correspond to a group of congenital heart diseases, with different anatomical combinations that culminate in a common characteristic: a single ventricle responds by both the systemic and pulmonary circulations. In most cases, it is possible to confirm the presence of two distinct ventricles, right and left, one being hypoplastic and called rudimentary and the other being well formed and called the main chamber.

One of the most common anatomic forms is the tricuspid atresia, with different presentations, which is responsible for 1.5-3% of congenital heart diseases and is present in approximately 0.6 out of every 10,000 live births. Another dominant left ventricular presentation is the pulmonary atresia, with intact interventricular septum, which accounts for about 1% of congenital heart diseases, occurring in about 0.7 out of every 10,000 newborns. Regarding to the dominant right ventricle, the main anatomical representation is the hypoplastic left heart syndrome (HLHS), which represents about 2% of congenital heart diseases, being present in approximately four out of every 10,000 newborns.

The natural history of single ventricular hearts is quite unfavorable: 64% of the patients were diagnosed during childhood and 50% in the neonatal period.

Treatment is usually performed through surgical staging, with interventions beginning in the neonatal period and culminating in the completion of a total cavopulmonary operation (Fontan)^[^^1^^]^, as a final stage.

The total cavopulmonary bypass allows close-to-normal saturation to these patients, decreasing the demand of the systemic ventricle. Despite this, it maintains altered hemodynamics leading to a series of late complications, among them, physical capacity reduction, ventricular dysfunction, intra- or extracardiac thrombi, arrhythmias, protein-losing enteropathy (PLE), and cirrhosis.

We carried out this study in order to evaluate the complications presented in the population submitted to total cavopulmonary bypass, with late follow-up at our institution, and to assess factors that may predispose to these complications, in addition to estimate the reintervention-free survival.

### Primary Goals

- To evaluate the late evolution of patients submitted to total cavopulmonary derivation with extracardiac conduit, with respect to complications (thrombi, PLE, ventricular and/or valvular dysfunction) presented and the need for reintervention.
- To evaluate factors that may interfere in the occurrence of complications or the need for reintervention: diagnosis, age of total cavopulmonary bypass, pulmonary vascular resistance and pressure, use of acetylsalicylic acid (ASA) or oral anticoagulation, and fenestration.

### Secondary Goal

- To evaluate signs of congestion and/or hepatic parenchyma alteration after five years of total cavopulmonary shunt through abdominal ultrasonography (USG).

## METHODS

This is a retrospective study using a chart analysis of 59 patients followed-up at the Single Ventricle Heart outpatient clinic of the Hospital do Coração (São Paulo, Brazil). To do so, we considered all patients undergoing total cavopulmonary bypass between 2000 and 2014. Patients with early death (in-hospital) and those under ambulatory follow-up at other institutions were excluded.

Anatomical and surgical data were obtained through a medical record survey conducted during in-hospital period and then outpatient follow-up.

Regarding the data of the surgical moment, we evaluated: diagnosis, staging performed, age of total cavopulmonary derivation, fenestration, pulmonary artery angioplasty/stenting, preoperative pulmonary pressure, preoperative pulmonary strength, and need for reintervention.

Concerning the late follow-up, we evaluated the occurrence of main late complications, such as: thrombi, arrhythmias, ventricular dysfunction, PLE, plastic bronchitis, and changes in hepatic parenchyma through abdominal USG.

Considering main late complications, we tried to identify factors that could interfere in the occurrence of complications and reintervention-free survival.

Initially all variables were analyzed descriptively. Analysis of quantitative variables was done by observing the minimum and maximum values and calculating means, standard deviations and median. Absolute and relative frequencies were calculated for qualitative variables. To test the homogeneity between proportions, chi-square test^[^^2^^]^ or Fisher's exact test was used.

The study of time until reintervention was performed through Kaplan-Meyer curve with log-rank test^[^^2^^]^. The software used for the calculations was SPSS 17.0 for Windows(r).

The level of significance used for the tests was 5%.

## RESULTS

During the study (from 2000 to 2014), 150 total cavopulmonary shunt operations were performed with an extracardiac conduit. Of these, 59 patients were followed-up at the Single Ventricular Heart outpatient unit of the Hospital do Coração.

These patients' ages varied from 10 to 186 months (mean of 4.45 years, standard deviation of 28.91 months, and median of 45 months), being 40 (70.2%) males and 17 (29.8%) females.

At the time of the study, follow-up time ranged from 10 days to 145 months (mean of 48.81 months, standard deviation of 36.68 months, and median of 37.37 months).

The distribution of patients being followed-up at our institution by diagnosis is detailed in Table 1. Thirty (50.8%) patients had left single ventricle and 29 (49.2%) had right single ventricle (48.2% of these patients presented with HLHS).

**Table 1:** Distribution of patients by diagnosis.

Diagnosis	Number of Patients (59)	Percentage (100%)
HLHS	14	23.7%
DILV with non-committed ventricular septal defect	3	5.1%
DORV with hypoplastic left heart and aortic arc	3	5.1%
Tricuspid atresia	11	18.6%
Mitral atresia	3	5.1%
DILV	10	16.9%
Pulmonary atresia with intact ventricular septum (PAIVS)*	7	11.9%
Right unbalanced atrioventricular septal defect	3	5.1%
Miscellaneous - Right SV	3	5.1%
Miscellaneous - Left SV	2	3.4%

* It includes one patient with critical pulmonary stenosis and an interventricular septum (functional pulmonary atresia with an interventricular septum with right ventricle hypoplasia) DILV=double inlet left ventricle; DORV=double outlet right ventricle; HLHS=hypoplastic left heart syndrome; PAIVS=pulmonary atresia with intact ventricular septum; SV=single ventricle

Among the patients with HLHS, 13 were submitted to the hybrid procedure (branch pulmonary arteries banding and stent implantation in ductus arteriosus) and only one was submitted to Norwood-Sano reconstruction as the first stage.

Fenestration between the extracardiac conduit and the right atrium was performed in 22 (37.2%) patients and the mean pulmonary artery pressure (MPAP) evaluated by preoperative catheterization was > 15 mmHg in 20 (33.8%) patients.

**Fig. 1:**
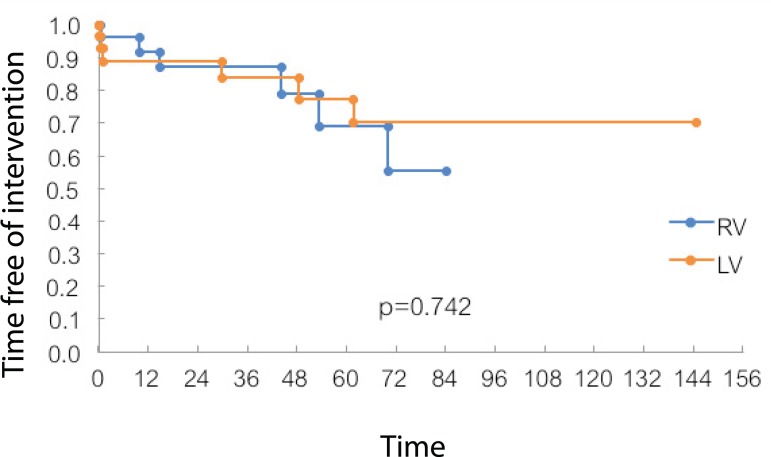
Time of follow-up free of reinterventions. LV=left ventricle; RV=right ventricle

Reintervention was necessary in 12 patients during follow-up for the reasons listed in Table 2. Among them, six patients had fenestration (*P*=0.31), two had a diagnosis of HLHS (*P*=1.00), and four had MPAP > 15 mmHg in the catheterization performed prior to total cavopulmonary bypass (*P*=1.00); none of these three factors could be related to the need for postoperative reintervention. When comparing it with the intervention-free evolution showed in Figure 1, it is observed that the curves are mixed, so the need for reintervention was not related to ventricular morphology in the study group.

**Table 2:** Interventions required during the postoperative period of total cavopulmonary shunt, considering the diagnosis and interval between total cavopulmonary shunt and reintervention.

Reintervention	Previous diagnosis	Time from total cavopulmonary derivation
Subaortic membrane resection and LV myectomy	DORV, ventricular septal defects (VSD)	15 months
Thrombus aspiration in EC and fenestration dilatation	HLHS	1 month
Tricuspid valve plastic, mitral valve closing and EC fenestration	DORV with left ventricular hypoplasia	69 months
Stent in pulmonary left artery and pulmonary right artery angioplasty with balloon catheter	PA with intact ventricular septum	29 days
Closing SPC and stent in pulmonary right artery	PA with intact ventricular septum	48 months
Surgical resection of infundibular septum and LV myectomy	DILV	61 months
Stent in pulmonary left artery	HLHS	43 months
Stent in pulmonary left and right arteries	CAVSD left unbalanced, right atrial isomerism, TAPVD, PA, aorta from RV	5 days
1) Occlusion of RVOT 2) Fenestration closing	Right single ventricle with double inlet right ventricle and DORV, TAPVD, right atrial isomerism	1) 13 days 2) 41 months
Fenestration dilatation and stent in pulmonary left and right arteries	HLHS	19 months
Tricuspid valve closing and reduction of fenestration	DILV	29 months
Stent in pulmonary left artery, stent in arterial branch to right lower lobe, and occlusion of RVOT	Critical pulmonary stenosis with intact ventricular septum and right ventricle hypoplasia	14 days

CAVSD=complete atrioventricular septal defects; DILV=double inlet left ventricle; DORV=double outlet right ventricle; EC=extracardiac conduit; HLHS=hypoplastic left heart syndrome; LV=left ventricle; PA=pulmonary atresia; RV=right ventricle; RVOT=right ventricular outflow tract; SPC=systemic-to-pulmonary collateral; TAPVD=total anomalous pulmonary venous drainage

**Fig. 2:**
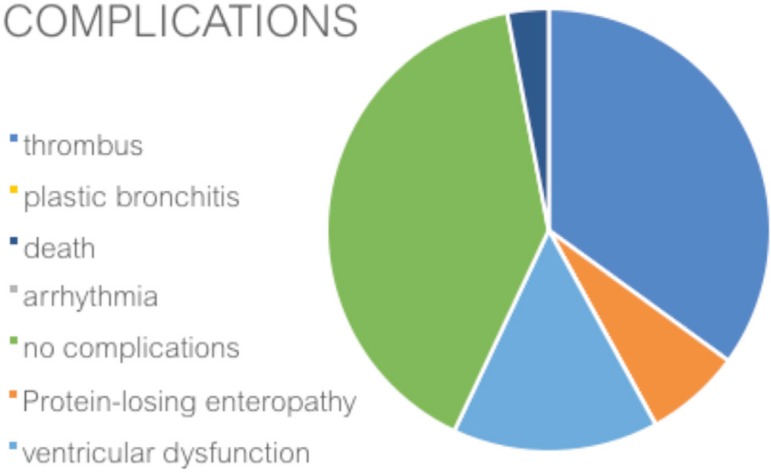
Occurrence of late complications (52%) in total cavopulmonary shunt

Among the patients evaluated, 31 (52%) presented with at least one of the complications considered; the complications found in late evolution are distributed as shown in Figure 2.

Two patients have died during the follow-up. However, among the 20 patients who presented with thrombosis at sometime of the follow-up, 50% of them were in the group of patients treated with anticoagulant therapy (*P*=0.54), the most common site being the extracardiac tube.

Regarding the PLE, it occurred in five patients (8.4%), two of them in the group with fenestrated tube (*P*=1.00); one patient had HLHS (*P*=1.00) and two patients were in the group with preoperative pulmonary pressure > 15 mmHg (*P*=0.6). Therefore, none of these factors can be related to the PLE occurrence in the postoperative period.

As secondary findings, ventricular dysfunction was present on echocardiogram in nine (15%) patients, five of them had fenestration (*P*=0.49); 77% had a right ventricle (*P*=0.04) and two had pulmonary pressure > 15 mmHg (*P*=1.00). Right ventricular morphology was the only factor that alone had statistically significant relationship with ventricular dysfunction in the outpatient follow-up of these patients.

In our institution, it is part of the postoperative follow-up protocol of patients submitted to total cavopulmonary bypass the abdominal USG examination at the postoperative fifth year to search for parenchymal alterations and/or signs of congestion justifying further investigation of possible hepatic cirrhosis. Among patients currently undergoing follow-up, 20 (35%) have been evaluated by USG and 10 (50%) presented with some degree of hepatic congestion and/or hepatomegaly. Between patients with this alteration, 16.7% of them had HLHS (*P*=0.057), 60% presented with pulmonary pressure > 15 mmHg and in 54% of them the tube was not fenestrated (*P*=1.00).

## DISCUSSION

Evolution and improvement in diagnosis, indication, surgical techniques, and postoperative care of patients with univentricular hearts have allowed an increasing survival rate of these patients, with quality of life quite close to normal. Thus, learning about the late complications to which this group of patients is subjected is fundamental. To improve care, it is essential to look at this group of patients in order to assess their demands.

Current late complications still very much resemble those found in recent years in the literature, even with the improvement of the technique proposed by Fontan in 1971^[^^1^^]^.

With the use of the technique based on extracardiac tube (technique used in all patients in this study), the difference in morbidity and mortality was mainly due to the occurrence of arrhythmias: the use of extracardiac tube to derive the flow from the inferior vena cava to the right pulmonary artery reduced significantly this occurrence^[^^3^^]^.

The use of the extracardiac polytetrafluoroethylene (PTFE) tube in all cavopulmonary bypass surgeries at our service without atrial scarring and without altering the atrium architecture justifies the absence of arrhythmias in the follow-up group at our center.

Currently, at the Hospital do Coração, all patients undergoing total cavopulmonary shunt are maintained using subcutaneous enoxaparin sodium, dose of 1 mg/kg/dose, 12/12 hours, until stabilization and possibility of oral anticoagulation. Oral anticoagulation is the prophylaxis of choice for thrombosis in these patients in the first two postoperative years and it is not performed in patients with social contraindication or drug intolerance. After two years, in the absence of episodes of thrombosis, all patients started to use ASA as prophylaxis, indefinitely. During follow-up, all patients underwent transesophageal echocardiography to evaluate the possibility of thrombi in the Fontan circuit, in five days, 90 days, 180 days, nine months, one year, 18 months, and two years postoperatively; after two years, they were evaluated once a year or whenever it was necessary. Thrombosis occurred in 35% of the patients, a higher occurrence than it is usually shown in the literature: 0-16% and 4.6%, according to some reports^[^^4^^]^, especially in the extracardiac tube and with no difference between the groups using ASA or oral anticoagulant, similar to a finding in a multicenter study published in 2011, which has found a 21% occurrence of thrombosis in the ASA group and 24% in the oral anticoagulant group^[^^5^^]^.

Another prospective study carried out in our department followed for two years a group of 40 patients submitted to cavopulmonary bypass, 20 in use of antiplatelet agent and 20 in use of oral anticoagulant, evidencing the absence of a statistically significant relation in the occurrence of thrombosis between the two groups, but showing a trend towards a greater deposit of material on the wall of the extracardiac tube, which was evidenced by computed tomography, in patients taking ASA^[^^6^^]^.

PLE is a serious event; its occurrence is found in most of the literature ranging from 1% to 5% of the patients and it has a 50% mortality rate in 5 years^[^^7^^,^^8^^]^.

Of the patients followed-up in our center, 7% developed with such complication. Among them, one required surgical intervention for the correction of major mitral valve insufficiency, with resolution of the condition. In a second case, we chose to perform a fenestration, and the child evolved to death postoperatively. Since the beginning of the use of oral budesonide in our service for the treatment of PLE, all other patients had this condition resolved, maintaining a serum level of albumin > 3 g/dL.

Regarding the ventricular dysfunction, it is a constant concern in the late follow-up of patients with univentricular heart morphology, because in addition to the cardiac insufficiency manifested in patients with ventricular dysfunction of any nature, it may compromise the operation of the Fontan type circuit, leading to pulmonary venocapilar congestion. Its occurrence was higher in patients with single ventricle of right morphology in our series and because of this the concern to preserve this ventricle is greater since the beginning of the follow-up. A study based on records from Australia and New Zealand of patients undergoing Fontan surgery (1268 in 2015) showed 7% of patients on (angiotensin converting enzyme) ACE inhibitors in order to preserve normal function and 36% systolic or diastolic dysfunction of the single ventricle. The use prevailed in patients with right ventricular morphology (70%)^[^^9^^]^.

Among the patients currently under follow-up, 35% have already underwent abdominal USG and 50% of them have alterations characterized by hepatic congestion. Between those, 16.7% of the alterations are in patients with HLHS (P=0.057). Hepatic impairment has been widely studied these days, being found from congestion to liver fibrosis and cirrhosis, changes that are directly related to the time since total cavopulmonary derivation^[^^10^^,^^11^^]^.

Current studies have shown a concern in proposing a protocol to evaluate liver changes in relation to periodicity of computed tomography, magnetic resonance imaging, and liver biopsy in search for precursor changes of hepatocarcinoma^[^^12^^,^^13^^]^.

## CONCLUSION

Complications such as thrombi and PLE were present in the assessed group during outpatient follow-up, but no notable relation with any of the pre or intraoperative factors assessed was found, so it remains a challenge to establish the best way to prevent and treat such complications in patients undergoing total cavopulmonary shunt.

No patient so far has evolved with arrhythmias or plastic bronchitis. Among the patients in follow-up, two had death as an outcome.

Among the evaluated causes that could interfere in the complications found, only the right ventricular morphology was statistically significant with ventricular dysfunction.

Regarding the hepatic congestion, the abdominal USG has shown signs of hepatic congestion in 50% of the evaluated cases, and a significant portion of them has been diagnosed with HLHS.

In order to evaluate parenchymal alterations, the indication of computed tomography and liver biopsy may be considered in patients with sign of congestion.

It is important to consider a better evaluation throughout this follow-up, trying to identify tissue changes, which are possibly precursors of hepatocellular carcinoma

**Table:** 

Authors' roles & responsibilities	
CFXP	Substantial contributions to the conception or design of the work; or the acquisition, analysis, or interpretation of data for the work; drafting the work or revising it critically for important intellectual content; final approval of the version to be published
PRSMC	Substantial contributions to the conception or design of the work; or the acquisition, analysis, or interpretation of data for the work; final approval of the version to be published
NFJB	Substantial contributions to the conception or design of the work; or the acquisition, analysis, or interpretation of data for the work; final approval of the version to be published
TMC	Agreement to be accountable for all aspects of the work in ensuring that questions related to the accuracy or integrity of any part of the work are appropriately investigated and resolved; final approval of the version to be published
IBJ	Agreement to be accountable for all aspects of the work in ensuring that questions related to the accuracy or integrity of any part of the work are appropriately investigated and resolved; final approval of the version to be published
